# A bypass mechanism of abiraterone‐resistant prostate cancer: Accumulating CYP17A1 substrates activate androgen receptor signaling

**DOI:** 10.1002/pros.23799

**Published:** 2019-04-24

**Authors:** Jan M. Moll, Jinpei Kumagai, Martin E. van Royen, Wilma J. Teubel, Robert J. van Soest, Pim J. French, Yukio Homma, Guido Jenster, Ronald de Wit, Wytske M. van Weerden

**Affiliations:** ^1^ Department of Urology Erasmus University Medical Center, Erasmus MC Cancer Institute Rotterdam The Netherlands; ^2^ Department of Urology University of Tokyo Tokyo Japan; ^3^ Department of Pathology Erasmus University Medical Center, Erasmus MC Cancer Institute Rotterdam The Netherlands; ^4^ Department of Erasmus Optical Imaging Centre Erasmus University Medical Center, Erasmus MC Cancer Institute Rotterdam The Netherlands; ^5^ Department of Cancer Treatment Screening Facility Erasmus University Medical Center, Erasmus MC Cancer Institute Rotterdam The Netherlands; ^6^ Department of Neurology Erasmus University Medical Center, Erasmus MC Cancer Institute Rotterdam The Netherlands; ^7^ Department of Medical Oncology Erasmus University Medical Center, Erasmus MC Cancer Institute Rotterdam The Netherlands

**Keywords:** abiraterone resistance, androgen receptor activation, castration‐resistant prostate cancer, cytochrome P450, family 17, subfamily A, polypeptide 1 inhibitor, TAK700

## Abstract

**Background:**

Intratumoral steroidogenesis and its potential relevance in castration‐resistant prostate cancer (CRPC) and in cytochrome P450, family 17, subfamily A, polypeptide 1 (CYP17A1)‐inhibitor treated hormone‐naïve and patients with CRPC are not well established. In this study, we tested if substrates for de novo steroidogenesis accumulating during CYP17A1 inhibition may drive cell growth in relevant preclinical models.

**Methods:**

PCa cell lines and their respective CRPC sublines were used to model CRPC in vitro. Precursor steroids pregnenolone (Preg) and progesterone (Prog) served as substrate for de novo steroid synthesis. TAK700 (orteronel), abiraterone, and small interfering RNA (siRNA) against *CYP17A1* were used to block CYP17A1 enzyme activity. The antiandrogen RD162 was used to assess androgen receptor (AR) involvement. Cell growth was measured by 3‐(4,5‐dimethylthiazol‐2‐yl)‐2,5‐diphenyltetrazolium bromide assay. AR‐target gene expression was quantified by reverse transcription polymerase chain reaction (RT‐PCR). Nuclear import studies using cells with green fluorescent protein (GFP)‐tagged AR were performed to assess the potential of precursor steroids to directly activate AR.

**Results:**

Preg and Prog stimulated cell proliferation and AR target gene expression in VCaP, DuCaP, LNCaP, and their respective CRPC sublines. The antiandrogen RD162, but not CYP17A1 inhibition with TAK700, abiraterone or siRNA, was able to block Preg‐ and Prog‐induced proliferation. In contrast to TAK700, abiraterone also affected dihydrotestosterone‐induced cell growth, indicating direct AR binding. Furthermore, Prog‐induced AR translocation was not affected by treatment with TAK700 or abiraterone, while it was effectively blocked by the AR antagonist enzalutamide, further demonstrating the direct AR activation by Prog.

**Conclusion:**

Activation of the AR by clinically relevant levels of Preg and Prog accumulating in abiraterone‐treated patients may act as a driver for CRPC. These data provide a scientific rationale for combining CYP17A1 inhibitors with antiandrogens, particularly in patients with overexpressed or mutated‐AR.

**Abbreviations**
AR: androgen receptor
CRPC: castration‐resistant prostate cancer
CYP17A1: cytochrome P450, family 17, subfamily A, polypeptide 1
DCC: dextran‐coated charcoal‐stripped fetal calf serum
DHT: dihydrotestosterone
FCS: fetal calf serum
MTT: 3‐(4,5‐dimethylthiazol‐2‐yl)‐2,5‐diphenyltetrazolium bromide
PC: prostate cancer
Preg: pregnenolone
Prog: progesterone
qPCR: quantitative PCR

## 1 INTRODUCTION

Castration‐resistant prostate cancer (CRPC) continues to rely on androgen receptor (AR) signaling for its growth, evidenced by the majority of patients with CRPC still responding to novel AR signaling pathway targeted agents. Both the antiandrogen enzalutamide (Xtandi),^1^, ^2^ the specific inhibitor of the steroidogenic enzyme cytochrome P450c17 (encoded by cytochrome P450, family 17, subfamily A, polypeptide 1 [*CYP17A1*]), abiraterone acetate (Zytiga), a 17‐α hydroxylase and 17,20‐lyase specific enzyme inhibitor blocking steroidal synthesis from androgen precursors^3^, ^4^ demonstrated survival benefit in patients with CRPC both in the pre‐ and in postdocetaxel treatment settings. Moreover, two recently published trials have reported benefit by combining abiraterone acetate with androgen deprivation therapy (ADT) vs ADT alone in metastatic hormone‐sensitive prostate cancer.^5^, ^6^ In contrast, the selective 17,20‐lyase inhibitor TAK700 (Orteronel])^7^ failed to demonstrate a survival benefit in either the pre‐ or postdocetaxel setting.^8^, ^9^ With novel hormonal agents now becoming the mainstay of advanced prostate cancer (PC) therapy in both hormone‐naïve and castration‐resistant setting, it is of importance to identify resistance mechanisms to these agents.

Clinical data have shown that a subgroup of patients with CRPC progressing on abiraterone still responds to enzalutamide,^10^ suggesting that the AR signaling axis is still active in these patients despite low circulating androgen levels.^11^ Several hypotheses have been postulated on the origin of AR reactivation in CRPC and CYP17A1‐inhibitor resistant disease. Indeed, Chen et al^12^ found the T877A AR mutation, rendering the AR activatible to progestagens, in 3 of 18 clinical abiraterone‐resistant CRPC samples. Romanel et al^13^ showed that not only AR gene modifications but also wild‐type AR copy number gain were associated with poor response to abiraterone and impaired overall survival. In addition, expression of the ligand‐independent AR variant V7 has been associated with a poor response to both enzalutamide and abiraterone.^14^

Preclinical studies have postulated CYP17A1‐dependent intratumoral de novo steroid synthesis as a driver of CRPC and CYP17A1‐inhibitor resistant disease. These studies reported de novo dihydrotestosterone (DHT) synthesis in LNCaP and VCaP cell lines^15^, ^16^, ^19^ and reduced AR target gene expression and DHT and T levels in CRPC xenograft tissue after abiraterone treatment.^18^ In contrast, we and others have found little evidence for de novo androgen synthesis in clinical CRPC samples. Moreover, we have previously shown that androgen precursors induced cell growth and AR target gene expression in vitro, but with undetectable CYP17A1‐dependent conversion into testosterone, indicating either direct AR binding or conversion rather than de novo synthesis as a driver of cell growth.^23^

In the present study, we assessed if CRPC cell growth could be driven by androgen precursors (pregnenolone [Preg] and progesterone [Prog]) at clinically relevant levels found in aging men^24^ as well as in patients treated with abiraterone.^25^, ^26^ We used CRPC models expressing wild‐type AR as well as mutated‐AR. Cell growth was studied in the presence of CYP17A1 enzyme‐ and AR‐inhibitors. To further establish the effects of precursor steroids on AR signaling, AR translocation was evaluated using a fluorescently labeled wild‐type AR.

## 2 MATERIALS AND METHODS

### 2.1 Cell culture

VCaP (a kind gift from Dr KJ Pienta, Baltimore, MD) and DuCaP (kindly provided and authenticated by Dr JA Schalken, Nijmegen, NL), both carrying wild‐type AR, were grown in Roswell Park Memorial Institute (RPMI) 1640 (Cambrex BioWhittaker, Wiesbaden, Germany) with 10% fetal calf serum (FCS) and antibiotics. Hep3B stably expressing the green fluorescent protein (GFP)‐AR^27^ were cultured in alpha minimum essential medium (Cambrex BioWhittaker) with 5% FCS, 2 mM l‐glutamine (Cambrex BioWhittaker), and antibiotics. PC346C (wild‐type AR) were maintained in prostate growth medium (PGM) based on Dulbecco's modified Eagle's medium (DMEM)‐F12 medium with several PC growth factors and antibiotics as described in Marques et al^28^ supplemented with 2% FCS (PAN Biotech, Aidenbach, Germany) and 0.1 nM of the synthetic androgen R1881 (NEN, Boston, MA). PC346 FLU1 (wild‐type AR) and PC346C FLU2 (AR^T877A^) cells were maintained in PGM supplemented with 2% dextran‐coated charcoal‐stripped FCS (DCC) instead of FCS, without R1881 and with the addition 1 μM of hydroxyflutamide. LNCaP cells (AR^T877A^; American Type Culture Collection [ATCC], Manassas, VA) were maintained in RPMI supplemented with 5% FCS and antibiotics. The adrenal cancer cell line H295R (ATCC) was maintained in DMEM‐F12 supplemented with 5% FCS and antibiotics. The cell lines PC346C, PC346C FLU1, and PC346C FLU2 were generated in our laboratory and are authenticated and described in Marques et al.^28^ PC346C‐GFP‐AR has been described in van Soest et al.^29^ For all experiments, cells were used within 6 months of resuscitation from cryopreservation. After completion of experiments, VCaP, LNCaP, and H295R were additionally verified by short tandem repeat genotyping using the Promega Powerplex 16 system (Madison, WI) in November 2014.

### 2.2 Establishment of CRPC cell lines

VCaP and DuCaP cells were cultured in RPMI with 10% DCC to deplete the serum from steroids in the presence of antiandrogens bicalutamide (1 μM) or hydroxyflutamide (1 μM), with n = 10 per condition for >20 months to generate a panel of castration‐resistant clones. When cell growth resembled that of the parental cells growing under standard culture conditions, clones were considered CRPC. For current experiments, clones expressing elevated levels of wild‐type AR and CYP17A1 compared with parental lines were selected for further experiments without further authentication.

### 2.3 Cell proliferation assays

For cell proliferation assays, 5,000 cells per well were plated in 96‐well dishes in their respective medium with DCC. After overnight attachment, the synthetic androgen R1881, steroids, and compounds were added to reach the indicated concentrations in a final volume of 200 μL. After 9 days, cell proliferation was assessed by 3‐(4,5‐dimethylthiazol‐2‐yl)‐2,5‐diphenyltetrazolium bromide (MTT)‐assay as described previously.^30^

### 2.4 Steroids and compounds

For cell culture assays, RD162, a nonsteroidal antiandrogen was used (Merck, Oss, Netherlands). It is closely related to and was selected from the same drug‐screen as MDV3100 (enzalutamide). In vitro and in vivo, it has equal potency in AR‐antagonism as enzalutamide and no significant difference in bioavailability in preclinical testing.^31^ For AR binding assays, enzalutamide (Axon Medchem, Groningen, The Netherlands) was used because of its current use in clinical practice.

Steroids were obtained from Steraloids (Newport, RI) and dissolved in ethanol. RD162, enzalutamide, TAK700 (Millennium Pharmaceuticals, Cambridge) or abiraterone (Johnson & Johnson, New Brunswick) were all dissolved in dimethyl sulfoxide (DMSO). Similar amounts of DMSO (0.1%) were added to control cells. Concentrations used were based on levels reported in Belanger et al^24^, ^25^ and Taplin et al^26^ (summarized in Table S1).

### 2.5 ∆4‐Androstenedione analysis

H295R cells at 100.000 cells per well were seeded into 24‐well tissue culture plates and allowed to attach overnight in medium with FCS, after which medium was replaced by the serum‐free medium with or without the *CYP17A1* inhibitors TAK700 (Millennium Pharmaceuticals) or abiraterone (Johnson & Johnson) for 48 hours. Medium from wells without cells served as blanks. Three replicates were used per condition. After 48 hours of culture, the medium was collected and frozen at −20°C. ∆4‐Androstenedione concentrations were determined using the IMMULITE 2000 automated assay system (Siemens DPC, Los Angeles, CA) with a detection limit of 1.05 nM. The results are shown as means ± SE of three independent experiments. Inhibitory concentration (IC50) values were determined by nonlinear regression using the GraphPad Prism software with *Y* = 100/(1 + 10^X−logIC50^).

### 2.6 *CYP17A1* knockdown

After overnight attachment, cells were transfected with CYP17A1 or nontargeting small interfering RNA (siRNA; On‐TARGETplus SMARTpool siRNA; Dharmacon, Lafayette, LA) using Lipofectamine RNAiMax (Invitrogen, Carlsbad, CA) according to the manufacturer's instructions. Twenty‐four hours after transfection, the medium was replaced by DCC medium with indicated steroids. RNA was isolated after 48 hours or proliferation determined at day 6.

### 2.7 Gene expression analysis

For quantitative polymerase chain reaction (qPCR) studies, RNA was isolated using RNA‐Bee (TEL‐TEST Inc, Friendswood, TX) from cultures treated for 48 hours with indicated compounds/steroids, 24 hours after seeding in DCC medium at 100.000 cells per well. Reverse transcriptase and qPCR runs were performed as described previously^21^ using an ABI Prism 7900 Sequence Detection System under standard conditions. Complementary DNA (cDNA; 20 ng) was amplified in SYBR Green PCR Master Mix (Applied Biosystems, Foster City, CA) or TaqMan Universal Master Mix (Applied Biosystems). PCR efficiency was verified by cDNA dilution curves and exceeding 90% for all assays. Primer/probe sets used are noted in Table S2. Gene expression was calculated as fold expression over housekeeping genes *GAPDH* or *PBGD* and vehicle treated cells.

### 2.8 Nuclear AR import studies

Nuclear translocation of the AR has been studied in time as well as in end‐point measurements using fluorescence confocal microscopy on PC346C cells stably expressing enhanced green fluorescent protein (EGFP)‐AR.^29^ To measure the effect of a concentration range of Preg and Prog, cells were seeded in a glass bottom 96‐well plate in culture medium supplemented with the charcoal‐stripped serum to avoid premature AR activation. Sixteen hours before imaging enzalutamide, TAK700, abiraterone (1 μM), and DMSO carrier only as control were added. Subsequently, 4 hours before imaging potential AR translocation was initiated using 0, 1, 10, and 100 nM Preg or Prog, and with 0.1 and 1 nM R1881 as the positive control, and nuclei were stained with Hoechst for reference. Cells were imaged using the Opera Phenix HCS system equipped with an x40 water immersion objective. Hoechst and EGFP were exited using 405 and 488 nm lasers and were visualized using 435 to 480 nm and 500 to 550 nm band‐pass filters. EGFP intensities were measured in the nuclear (nuc) and the peri‐nuclear (cyto) regions. Nuclear translocation of the AR was expressed by nuclear signal intensity/(nuclear signal intensity+cytoplasmatic signal intensity), after background subtraction.

The ratio of AR nuclear localization was expressed as:

For the analysis of AR‐translocation dynamics, cells were seeded on glass coverslips in six‐well plates. After overnight attachment, cells were treated with TAK700 (3 µM) or vehicle for 12 hours and subsequently transferred to a live‐cell chamber and maintained at 37°C and 5% CO_2_. Time‐lapse imaging was performed using a Zeiss LSM510 confocal microscope (Carl Zeiss, Jena, Germany), equipped with a 63 × 1.3 NA oil immersion objective. EGFP‐AR was visualized using 488 nm excitation of an Argon‐laser line and detection of emission between 500 and 530 nm. For time‐lapse imaging, images were acquired with 5 minutes interval during 130 minutes at multiple locations of the same sample. After 5 to 10 minutes of imaging, Prog (100 nM), Preg (100 nM), or R1881 (1 nM) was added to the medium to investigate AR nuclear translocation. Average fluorescence intensities in the nucleus and cytoplasm were measured at every time point.

### 2.9 Data analysis

MTT and qPCR results were normalized to control and compared using the two‐sided Student *t* test. AR translocation was analyzed using one‐way analysis of variance (ANOVA) with subsequent Tukey's multiple comparisons on logarithmically transformed values to equalize variances. Analyses were carried out using the GraphPad Prism version 5.03 (GraphPad Software, San Diego, CA). *P* < 0.05 was considered statistically significant.

## 3 RESULTS

### 3.1 *CYP17A1* inhibitors abiraterone and TAK700 effectively inhibit steroidogenesis in H295R cells

To determine IC50 values of the CYP17A1 inhibitors under the conditions used in this study, human adrenal H295R cells were incubated in steroid stripped medium with increasing amounts of abiraterone or TAK700. ∆4‐Androstenedione synthesis—which is directly dependent on CYP17A1 activity (Figure S1A)—was effectively blocked in H295R cells (expressing CYP17A1 1100‐fold vs prostate cells) with IC50 values of 15.5 (95% confidence interval [CI], 10.4‐23.0) and 67.7 (95% CI, 58.6‐78.1) nM for abiraterone and TAK700, respectively (Figure S1B).

### 3.2 Preg‐ and Prog‐mediated cell growth of CRPC clones is independent of *CYP17A1* enzymatic activity

**Figure 1:**
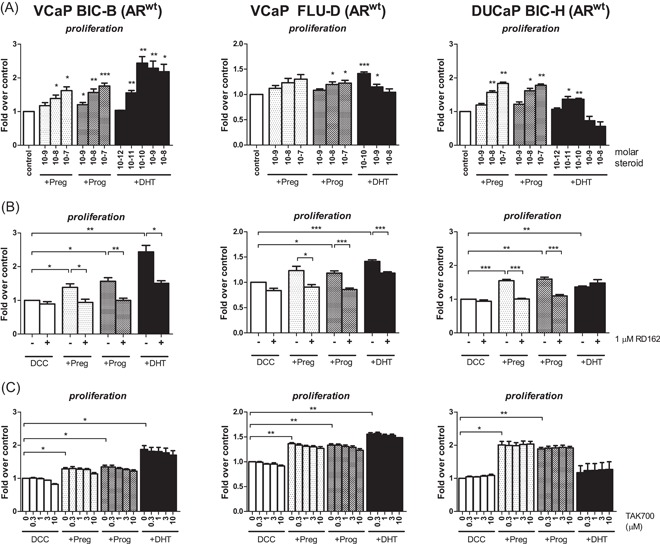
AR‐blockade, but not CYP17A1 inhibition reduced Preg‐ and Prog‐induced cell growth in CRPC clones of VCaP and DuCaP. A, VCaP CRPC derivatives BIC‐B and FLU‐D and DuCaP CRPC derivative BIC‐H were plated in DCC medium and incubated with vehicle (ethanol, white), Preg (light gray), Prog (dark gray), or DHT (black) at the indicated levels. Data are expressed as mean ± SE of three independent experiments. DuCaP BIC‐H appeared hypersensitive to androgens, growing even better with Preg or Prog as compared with DHT. B, VCaP BIC‐B and FLU‐D and DuCaP BIC‐H were incubated with 10 nM of Preg or Prog, or with 0.1 nM DHT with or without 1 μM of the antiandrogen RD162. Data shown are mean ± SE of four independent experiments. C, VCaP BIC‐B and FLU‐D and DuCaP BIC‐H were treated with 100 nM of Preg or Prog or with 0.1 nM DHT with or without TAK700 at the indicated concentrations. Data shown are mean ± SE of three independent experiments. *P < 0.05, **P < 0.01, ***P < 0.001. AR, androgen receptor; CRPC, castration‐resistant prostate cancer; CYP17A1, cytochrome P450, family 17, subfamily A, polypeptide 1; DCC, dextran‐coated charcoal‐stripped fetal calf serum; DHT, dihydrotestosterone; Preg, pregnenolone; Prog, progesterone

To assess the biological relevance of CYP17A1‐mediated cell growth in CRPC, we selected CRPC cell lines of VCaP (BIC‐B and FLU‐D, resistant to bicalutamide and flutamide, respectively) and DuCaP (BIC‐H) based on their elevated *CYP17A1* and *AR* gene expression level compared with their parental cell lines (Table S3). Preg and Prog at levels of 1 nM and upstimulated cell growth in all CRPC clones tested (Figure 1A). RD162 effectively blocked 10 nM Prog‐ and Preg‐induced cell growth (Figure 1B), indicating that the proliferative effects of Preg and Prog were AR‐driven. Despite complete CYP17A1 inhibition as demonstrated in H295R cells, TAK700 could not inhibit Preg‐ and Prog‐induced cell growth in these CRPC clones in concentrations up to 10 μM, which is approximately 150 times the IC50 in our in vitro conditions (Figure 1C). Preg‐ and Prog‐activated AR was confirmed by upregulated expression of the AR‐target gene *PSA* in VCaP BIC‐B even in the presence of TAK700 (Figure S2). Similar results were obtained for LNCaP (Figure S3), and PC346C CRPC cells that are characterized by an overexpressed wild‐type AR (PC346C FLU1) or by T877A AR mutation (PC346C FLU2; Figure S4). Of note, *CYP17A1* messenger RNA (mRNA) could not be detected in LNCaP nor in PC346C (Table S3). Also, we were unable to detect CYP17A1 protein in VCaP despite detectable mRNA levels. (Figure S5). The AR‐driven effects in the presence of TAK700 indicate that growth of these CRPC clones, despite upregulated *CYP17A1* mRNA, was not dependent on the increased activity of de novo steroidogenesis.

**Figure 2:**
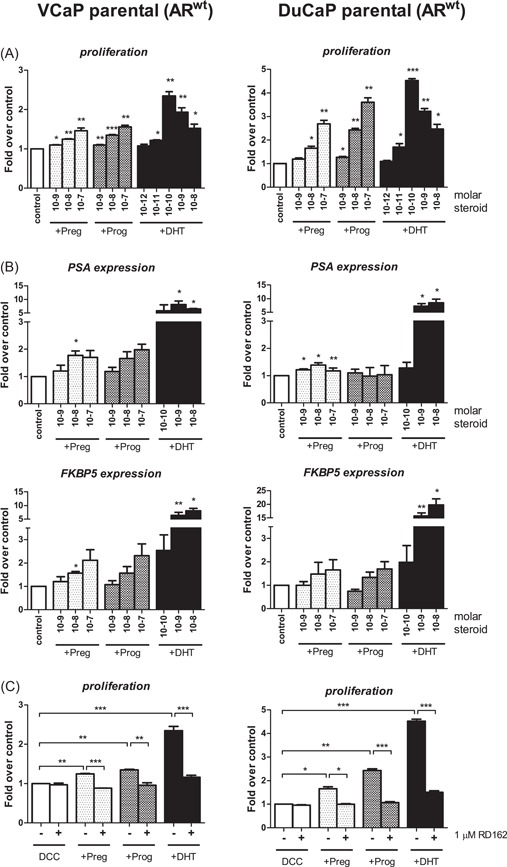
Preg‐ and Prog‐activated AR in VCaP and DuCaP. A, Castration‐naïve VCaP (left) and DuCaP (right) cells were treated with Preg (light gray), Prog (dark gray), or DHT (black) at indicated concentrations (M). Data shown are mean ± SE of three independent experiments. B, Castration‐naïve VCaP (left) and DuCaP (right) cells were treated with Preg or Prog or DHT for 48 hours at indicated concentrations (M), and gene expression was assessed by qPCR with each sample in duplicate. Data shown are mean ± SE of three independent experiments. C, VCaP and DuCaP cells were treated with 100 nM Preg or Prog of 0.1 nM with or without RD162. Cell growth was assessed by MTT assay on day 9. Data shown are mean ± SEM of a minimum of three independent experiments. *P < 0.05, **P < 0.01, ***P < 0.001. AR, androgen receptor; CYP17A1, cytochrome P450, family 17, subfamily A, polypeptide 1; DCC, dextran‐coated charcoal‐stripped fetal calf serum; DHT, dihydrotestosterone; Preg, pregnenolone; Prog, progesterone; qPCR, quantitative polymerase chain reaction

**Figure 3:**
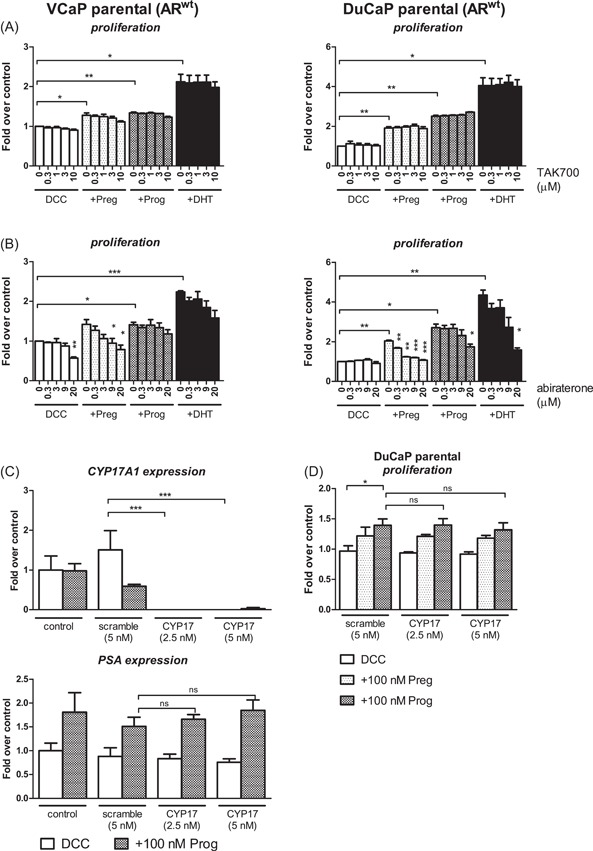
Preg‐ and Prog‐induced cell growth of castration‐naïve VCaP and DuCaP is independent of CYP17A1 activity. Cells were treated with 100 nM of Preg (light gray) or Prog (dark gray), or 0.1 nM DHT (black) with or without TAK700 (A) or abiraterone (B) at indicated concentrations. Data shown are mean ± SE of three independent experiments. C, VCaP cells were transfected with 2.5 or 5 nM *CYP17A1*‐directed or 5 nM scrambled siRNA (Dharmacon) for 24 hours, after which cells were incubated with vehicle or 100 nM Prog for 48 hours after which *CYP17A1* and *PSA* mRNA expression were assessed by qPCR with each sample in duplicate. D, DuCaP cells were transfected with 2.5 or 5 nM *CYP17A1* siRNA or 5 nM scrambled siRNA (Dharmacon) for 24 hours and subsequently incubated with Preg, Prog, or vehicle (ethanol) for 6 days. Cell proliferation was assessed by MTT assay. Data shown are mean ± SE of three independent experiments. *P < 0.05, **P < 0.01, ***P < 0.001. AR, androgen receptor; *CYP17A1*, cytochrome P450, family 17, subfamily A, polypeptide 1; DCC, dextran‐coated charcoal‐stripped fetal calf serum; DHT, dihydrotestosterone; mRNA, messenger RNA; Preg, pregnenolone; Prog, progesterone; qPCR, quantitative polymerase chain reaction; siRNA, small interfering RNA

Prog and Preg stimulate cell growth of castration‐naïve VCaP and DuCaP cells via AR activation to test if Preg and Prog could also facilitate cell growth of castration‐naïve parental VCaP and DuCaP, characterized by relatively low levels of CYP17A1 (Table S3), cells were incubated with 10 and 100 nM Prog and Preg. Indeed, cell growth was significantly stimulated (Figure 2A) with concomitant induction of AR target gene expression, although to a lesser extent than by DHT (Figure 2B). One micrometer RD162 significantly blocked Prog‐ and Preg‐induced VCaP and DuCaP cell growth (Figure 2C), substantiating that the proliferative effect of Preg and Prog were AR‐driven. Furthermore, TAK700 did not inhibit Preg‐ or Prog‐induced cell growth, indicating the effect to be independent of CYP17A1 activity also in hormone‐naïve PC cell lines (Figure 3A). Abiraterone affected Preg‐stimulated but not Prog‐induced cell proliferation, and only at concentrations exceeding the IC50 for CYP17A1 inhibition that also blocked DHT‐induced cell growth (Figure 3B)

To further substantiate that cell growth in hormone‐naive PC cells is independent of CYP17A1, VCaP, and DuCaP were treated with siRNA for *CYP17A1*. Similarly to incubation with TAK700, treatment with *CYP17A1* siRNA—resulting in undetectable levels of *CYP17A1* mRNA—did not affect Prog stimulated levels of *PSA* mRNA in parental VCaP (Figure 3C). Likewise, in DuCaP, Preg‐ and Prog‐induced cell proliferation was unaffected by *CYP17A1‐*directed siRNA (Figure 3D).

### 3.3 Preg and Prog translocate AR to the nucleus without requiring conversion into testosterone

**Figure 4:**
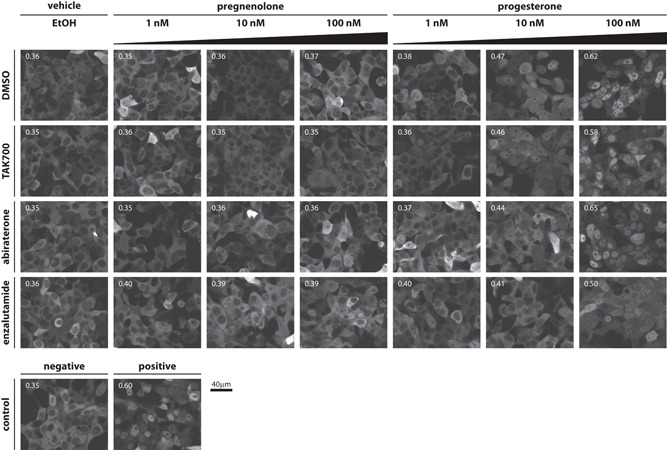
CYP17A1 inhibitors TAK700 and abiraterone are unable to inhibit progesterone‐induced AR translocation. Top, representative images of PC346C‐GFP‐AR cells 3 hours after the addition of vehicle control (ethanol) or increasing levels of Preg or Prog, with each row showing representative images after pretreatment with either DMSO, 1 μM TAK700, 1 μM abiraterone or 1 μM enzalutamide. Bottom, representative images 3 hours after addition of negative control (ethanol+DMSO) or positive control: 1 nM R1881+DMSO. Scale bar = 40 μm. The number in the top‐left of each picture indicates average nuclear/total AR signal ratio. Corresponding graph bars with SD are provided in Figure S7. AR, androgen receptor; CYP17A1, cytochrome P450, family 17, subfamily A, polypeptide 1; DMSO, dimethyl sulfoxide; GFP, green fluorescent protein; Preg, pregnenolone; Prog, progesterone

To prove the direct effects of Preg and Prog on wild‐type AR, AR translocation to the nucleus was evaluated in Hep3B cells expressing GFP‐tagged‐AR^wt^. Incubation with 100 nM Prog resulted in direct AR translocation, despite preincubation with 3 μM TAK700 (~45 times the IC50 of CYP17A1 inhibition in H295R cells; Figure S6). To further substantiate these findings, we evaluated AR translocation in the human prostate cancer cell line PC346C, which naturally expresses AR^wt^ and lacks CYP17A1 expression. Incubation with Prog, but not Preg, induced translocation of AR to the nucleus in PC346C cells stably transfected with GFP‐AR^wt^. This translocation could not be inhibited by overnight preincubation with 1 μM TAK700 or abiraterone, but only by pretreatment with 1 μM of the AR antagonist enzalutamide (Figure 4), indicating that these effects were indeed dependent on direct activation of AR.

## 4 DISCUSSION

Here, we show that clinically relevant levels of androgen precursors Preg and Prog stimulated cell growth of parental VCaP and DuCaP and of their respective CRPC cell lines that are characterized by overexpressed levels of wild‐type *AR* and *CYP17A1*. These precursors have been shown to commonly accumulate in patients during CYP17A1 therapy. This growth induction could be effectively blocked by the potent AR antagonist RD162, but not by CYP17A1‐specific inhibition by TAK700, abiraterone or by siRNA. These cell growth effects were paralleled by the induction of AR target gene expression, indicating these effects were AR‐driven. Similar results were observed in LNCaP and the flutamide‐resistant CRPC cell line PC346 FLU2, which both carry the T877A mutation in the ligand‐binding domain of the AR, but also in the flutamide‐resistant CRPC cell line PC346 FLU1, which overexpresses wild‐type AR. Together, these results suggest a common mechanism of androgen precursor‐induced cell growth in AR‐overexpressing CRPC that drives cell growth independent of de novo androgen synthesis through CYP17A1, but via direct AR‐stimulation. Our AR nuclear import studies further supported Preg and Prog to be able to directly activate wild‐type AR. These data provide an alternative mechanism of (CYP17A1‐induced) CRPC resistance that is driven by accumulating precursor androgens that may directly mediate AR‐regulated cell growth, particularly in tumors overexpressing AR. Indeed, recent studies on cell‐free DNA in patients with metastatic CRPC have demonstrated that AR copy number gain at the initiation of treatment with second‐line hormonal agents is indicative of primary resistance to these agents.^13^, ^32^

Data on precursor steroid levels in patients treated with abiraterone are scarce. As a surrogate measure of systemic steroid concentrations, urinary Preg and Prog metabolites in patients treated with abiraterone without exogenous glucocorticoids have previously been shown reported to be 2.5 to 44 and 3.8 to 61 times higher compared with baseline.^25^ In patients treated with concomitant glucocorticoids, serum levels of Preg and Prog decreased markedly (Table S1). Taplin et al^26^ reported intraprostatic steroid tissue concentrations from prostate biopsies in patients undergoing neoadjuvant castration in combination with abiraterone and prednisone before radical prostatectomy. As expected, intratumour androgen concentrations decreased dramatically, but with the consequence of increasing levels of the CYP17A1 substrates Preg and Prog (mean for Preg, 142 nM; Prog, 1 nM). These levels were comparable to levels that demonstrated growth induction in our CRPC models.

With CYP17A1 expression being a magnitude lower in prostate cells compared with adrenal cells (Figure S4 and Luu ‐The et al^33^) and TAK700 and abiraterone inhibiting CYP17A1 in human adrenal H295R cells at 67 and 15 nM, respectively, the steroid synthesis inhibitor levels used in this study should effectively inhibit CYP17A1 activity in prostate cells. The fact that neither clinically relevant levels of TAK700 (up to 6 μM^34^), abiraterone (up to 2 μM^35^) nor siRNA against *CYP17A1* were able to reverse Preg‐ or Prog‐induced cell growth of CRPC at clinically relevant levels shown in our study underscore reports that intratumoral de novo steroidogenesis is not essential for CRPC growth.^36^

We demonstrate that inhibition of AR‐mediated cell growth by abiraterone can be (partly) explained by direct AR‐antagonism, albeit at peak concentrations. This is consistent with data that abiraterone can bind to and antagonize AR in LNCaP and VCaP,^37^, ^38^ and our prior report that abiraterone can also partially block AR nuclear translocation.^29^ Furthermore, the previously observed 3βHSD inhibition,^39^ combined with the antiandrogenic potency and inhibition of CYP17A1, 3βHSD, and SRD5A by the abiraterone metabolite, D4‐abiraterone,^40^ may explain the beneficial effect of abiraterone in clinical trials relative to TAK700, which seems to lack these additional effects.

To date, two retrospective studies reported PSA response rates of 27% and 30% for enzalutamide in abiraterone progressive patients.^41^, ^42^ Interestingly, in a third retrospective study, PSA responses for enzalutamide after abiraterone vs abiraterone after enzalutamide have been reported to be higher (30% vs 6%), with a trend towards longer PFS in the first group.^43^ In contrast, adding abiraterone to continued enzalutamide treatment did not result in a significant delay of PSA progression vs abiraterone alone in patients with biochemical progression on enzalutamide.^44^ However, none of these studies have interrogated the upfront combination of AR‐antagonism with androgen synthesis inhibition.

Although our study may be limited by the use of in vitro models, it is important to note that these different cell lines were selected based on highly clinically relevant characteristics, including the absence of intratumoral CYP17A1 expression in the context of high AR expression. The studies were performed using clinically relevant levels of steroids and drugs as reported from relevant patient cohorts, to recapitulate the negative consequences of enhanced substrate levels of preg and prog in patients with CRPC treated with CYP17A1 inhibitors. The in vitro system allows for defined assessment of the potential of different steroids that will otherwise be obscured in in vivo models. These data provide basic mechanistic evidence to combine steroid synthesis inhibition with antiandrogens to fully extinguish ligand‐dependent AR activation in tumors that have become hypersensitive to minute levels of androgen or alternative steroidal ligands (like accumulating progestagens due to systemic CYP17A1 inhibition) via AR amplification or mutations in the absence of intratumoral CYP17A1 activity. Thus, prospective data on PSA response and possible survival benefit of combining abiraterone with enzalutamide from the start of second‐line hormonal therapy are eagerly awaited.^45^

## 5 CONCLUSIONS

**Figure 5:**
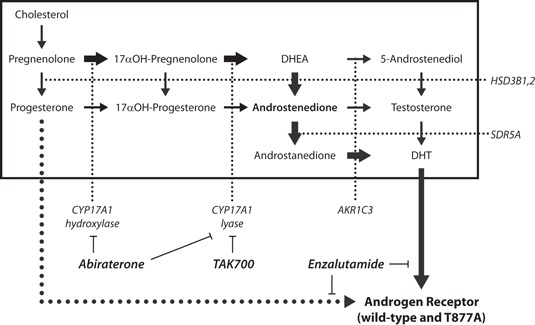
Schematic representation of alternative mechanisms of CRPC. Simplified overview of the classical androgen synthesis pathway. Thick arrows indicate the preferred steps in human androgen biosynthesis and subsequent AR activation as reported in the literature. Abiraterone and TAK700 effectively inhibit steroid synthesis, but cannot prevent direct binding of the steroid precursors Preg or Prog to the AR (dotted line). Direct AR‐antagonism by enzalutamide will still block activation by either DHT or Prog. AR, androgen receptor; CRPC, castration‐resistant prostate cancer; *CYP17A1*, cytochrome P450, family 17, subfamily A, polypeptide 1; DHT, dihydrotestosterone; Preg, pregnenolone; Prog, progesterone

In summary, our study demonstrates that in castration‐naïve and CRPC cell lines, androgen precursor steroids Preg and Prog are able to directly activate wild‐type and mutated‐AR, independent of CYP17A1‐mediated conversion into testosterone (Figure 5). These findings may indicate a mechanism of resistance for patients progressing on CYP17A1 therapy where enzyme inhibition causes accumulation of these androgen precursors, and provide an explanation of why CYP17A1‐inhibitor‐resistant tumors may still respond to treatment with antiandrogens. From a clinical perspective, these data support the rationale for the combination of CYP17‐inhibitors with potent antiandrogens, to effectively suppress AR activation mediated by accumulating steroidal ligands in both AR‐amplified and AR‐mutated tumors.

## CONFLICT OF INTERESTS

RW receives consultancy and speaker fees from Sanofi, Millennium, Merck, Roche. RS receives speaker fee from Sanofi, Janssen Pharmaceuticals. WW receives grant supports from Sanofi, Millennium, Janssen Pharmaceuticals, Servier.

## Supporting information

Supporting information

Supporting information

Supporting information

Supporting information

Supporting information

Supporting information

Supporting information

Supporting information

Supporting information

Supporting information

Supporting information
