# Supplementary material for: A bypass mechanism of abiraterone‐resistant prostate cancer: Accumulating CYP17A1 substrates activate androgen receptor signaling
Source: Prostate. 2019 Apr 24;79(9):937–48. doi: 10.1002/pros.23799 (PMC6593470; doi:10.1002/pros.23799)
Supplement: Supplementary file 5 — Supporting information [file PROS-79-937-s005.doc]

**Supplementary figure 5: CYP17A1 protein was readily detected in H295R adrenal cells, but not in VCaP parental or CRPC cells**

VCaP par

H295R

VCaP FLU-D

VCaP BIC-B

αCYP17A1

αAR

Antibodies used:
CYP17A1, sc-46084, SantaCruz
AR, clone SP107, Cell Marque
