# Supplementary material for: A bypass mechanism of abiraterone‐resistant prostate cancer: Accumulating CYP17A1 substrates activate androgen receptor signaling
Source: Prostate. 2019 Apr 24;79(9):937–48. doi: 10.1002/pros.23799 (PMC6593470; doi:10.1002/pros.23799)
Supplement: Supplementary file 8 — Supporting information [file PROS-79-937-s008.doc]

**Supplementary Figure Legends**

**Supplementary Figure 1. Inhibition of steroidogenesis in H295R cells by abiraterone and TAK700**

A. Overview of the classic androgen synthesis pathway. Large arrows represent the main conversions in humans. The black arrows indicate the conversions by CYP17A1. Abiraterone blocks both 17-α hydroxylase and 17,20-lyase, whereas TAK700 only inhibits CYP17A1 17,20-lyase activity. Production of ∆4-androstenedione requires both enzymatic conversions for its production.

B. Autonomous ∆4-androstenedione production in human adrenal cells. H295R cells were treated with abiraterone or TAK700 for 48 hr. ∆4-androstenedione levels were measured in supernatants by chemiluminescent immunoassay. Data were analyzed by nonlinear regression using GraphPad Prism to determine IC50 values for each compound. Data shown are mean ± SEM of three independent experiments.

**Supplementary Figure 2. Preg or Prog-induced PSA and FKBP5 mRNA expression which is not affected by TAK700 treatment.**

PSA (top) and FKBP5 (bottom) AR target gene expression in castration-naïve (left) and CRPC (right) VCaP cells. Cultures were treated for 48 hours with 100 nM of Preg, Prog, or 0.1 nM DHT with or without indicated concentrations of TAK700. Gene expression was assessed by qPCR with each sample in duplicate. Data are expressed as mean ± SE of two independent experiments.

**Supplementary Figure 3. Preg or Prog-induced cell growth of LNCaP is not blocked by TAK700**

LNCaP cells were treated with 100 nM of Preg or Prog, or 0.1 nM DHT with or without TAK700 at the indicated concentrations, with proliferation assessed by MTT assays at Day 9 with 4 replicates per condition. Data shown are mean ± SE of three independent experiments.

**Supplementary Figure 4. Induction of proliferation and AR-target gene expression in AR-overexpressing PC346C FLU1 and PC346C FLU2**

Cell proliferation and expression of AR target genes in castration-naïve PC346C (left) and CRPC clones PC346 FLU1 (center) and PC346C FLU2 (right).

Top: MTT proliferation assay results. In line with previous reports, PC346C did not respond to Preg or Prog. In PC346 FLU1, AR target gene expression was stimulated by 100 nM Prog. In PC346 FLU2, harboring the T887A mutation, proliferation could be stimulated from 10 nM Prog. MTT assays were carried out at Day 9 with 8 replicates per condition. Data are shown as mean ± SE of 3-4 independent experiments.

Middle: qPCR results for *PSA*. In cells overexpressing AR (FLU1) or having a mutant AR (FLU2), Preg and Prog induced *PSA* expression. Data are shown as mean ± SE of a 3-4 independent experiments.

Bottom: qPCR results for *FKBP5*. In cells overexpressing AR (FLU1) or having a mutant AR (FLU2), Preg and Prog induced *FKBP5* expression. Data are shown as mean ± SE of 3-4 independent experiments.
