# Supplementary material for: A bypass mechanism of abiraterone‐resistant prostate cancer: Accumulating CYP17A1 substrates activate androgen receptor signaling
Source: Prostate. 2019 Apr 24;79(9):937–48. doi: 10.1002/pros.23799 (PMC6593470; doi:10.1002/pros.23799)
Supplement: Supplementary file 9 — Supporting information [file PROS-79-937-s009.doc]

**Supplementary Table 1: Tumour and plasma s**teroid concentrations and changes in healthy volunteers and abiraterone-treated patients

| Steroid | Belanger et al [24] *plasma* | Attard et al [25]  *plasma* | | Taplin et al [26],  *tumor* |
| --- | --- | --- | --- | --- |
|  | Healthy 60 year old volunteers  (nM ) | Abiraterone treated patients w.o. dexamethasone,  % change v.s. baseline | Abiraterone treated patients after addition of dexamethasone,  % change v.s. no dexamethasone | LHRH agonist + abiraterone acetate + prednisone *vs* LHRH agonist alone  % change |
| Pregnenolone | 1.61 ± 0.02 | 842 | -93 | +692 |
| Progesterone | 0.49 ± 0.02 | 1919 | -91 | +365 |
| DHEA | 6.92 ± 0.10 | -97 | -43 | -98 |
| Androstenedione | 2.19 ± 0.03 |  |  | -77 |
| Testosterone | 10.12 ± 0.14 |  |  | -38 |
| DHT | 3.22 ± 0.05 | -94 | -39 | -86 |
