# Supplementary material for: A bypass mechanism of abiraterone‐resistant prostate cancer: Accumulating CYP17A1 substrates activate androgen receptor signaling
Source: Prostate. 2019 Apr 24;79(9):937–48. doi: 10.1002/pros.23799 (PMC6593470; doi:10.1002/pros.23799)
Supplement: Supplementary file 10 — Supporting information [file PROS-79-937-s010.doc]

**Supplementary T**able 2: Primer/probe sequences used

| *SYBR primersets* | | | |
| --- | --- | --- | --- |
| Gene | Forward primer | Reverse primer |  |
| *PBGD* | 5’-CATGTCTGGTAACGGCAATG-3’ | 5’-GTACGAGGCTTTCAATGTTG-3’ |  |
| *FKBP5* | 5’-GAATACACCAAAGCTGTTGA-3’ | 5’- CTCTTCCTTGGCATCCT-3’ |  |
| *TaqMan primer/probesets* | | | |
| Gene | Forward primer | Reverse primer | Probe |
| *AR* | 5’-CATCAAGGAACTCGATCGT-3’ | 5’-GAACTGATGCAGCTCTCTC-3’ | 5’-ACATCCTGCTCAAGACGCTCCT-3’ |
| *PSA* | 5’-CCCTCAGAAGGTGACCA-3’ | 5’-ACCACCTTGGTGTACAGG-3’ | 5’-TATCACGTCATGGGGCAGTG-3’ |
| Bought primer/probe sets (Life technologies, Bleiswijk, Netherlands) | | | |
| *GAPDH* | Hs99999905_m1 |  |  |
| *CYP17A1* | Hs01124136_m1 |  |  |
