# Supplementary material for: A bypass mechanism of abiraterone‐resistant prostate cancer: Accumulating CYP17A1 substrates activate androgen receptor signaling
Source: Prostate. 2019 Apr 24;79(9):937–48. doi: 10.1002/pros.23799 (PMC6593470; doi:10.1002/pros.23799)
Supplement: Supplementary file 11 — Supporting information [file PROS-79-937-s011.doc]

**Supplementary Table 3: Relative AR and CYP17A1 expression in VCaP, DuCaP, LNCaP, PC346C and CRPC derivatives**

| **Cell line** | **GAPDH** |  | **AR** |  |  | **CYP17A1** |  |
| --- | --- | --- | --- | --- | --- | --- | --- |
| **Parental** | Ct | Ct | ΔCt |  | Ct | ΔCT |  |
| VCaP | 24,17 | 19,12 | -5,05 |  | 38,39 | 14,22 |  |
| DuCaP | 24,42 | 24,25 | -0,17 |  | nd | na |  |
| LNCaP | 23,10 | 21,20 | -1,90 |  | nd | na |  |
| PC346C | 22,30 | 22,25 | -0,05 |  | nd | na |  |
| **CRPC** | Ct | Ct | ΔCt | Fold expression vs parental* | Ct | ΔCT | Fold expression vs parental* |
| VCaP BIC-B | 23,02 | 18,44 | -4,58 | 0,72 | 34,43 | 11,41 | 7,01 |
| VCaP FLU-D | 23,15 | 17,99 | -5,16 | 1,08 | 34,62 | 11,47 | 6,73 |
| DuCaP BIC-H | 23,96 | 18,52 | -5,44 | 38,59 | 38,70 | 14,74 | na |
| PC346C Flu1 | 21,78 | 19,57 | -2,21 | 4,47 | nd | na | na |
| PC346C Flu 2 | 25,34 | 25,43 | 0,09 | 0,91 | nd | na | na |

* based on ΔΔCT
